# Superior Capsular Reconstruction of the Shoulder Using the Long Head of the Biceps Tendon: A Systematic Review of Surgical Techniques and Clinical Outcomes

**DOI:** 10.3390/medicina57030229

**Published:** 2021-03-02

**Authors:** Dimitrios Kitridis, Christos Yiannakopoulos, Chris Sinopidis, Panagiotis Givissis, Nikiforos Galanis

**Affiliations:** 1 1st Orthopaedic Department, School of Medicine, Aristotle University of Thessaloniki, 54124 Thessaloniki, Greece; christos.sinopidis@gmail.com (C.S.); pgivissis@gmail.com (P.G.); ngalanismed@gmail.com (N.G.); 2 School of Physical Education and Sports Science, National and Kapodistrian University of Athens, 15772 Athens, Greece; ckyortho@gmail.com

**Keywords:** shoulder, arthroscopy, rotator cuff, superior capsular reconstruction, long head of biceps

## Abstract

*Background and Objectives:* Superior capsular reconstruction (SCR) with the use of a fascia lata autograft or a dermal allograft is an established treatment in treating irreparable rotator cuff (RC) tears. The long head of the biceps tendon (LHBT) has been recently proposed as an alternative graft for SCR. The purpose of this study was to present the surgical techniques and clinical studies utilizing the LHBT for SCR. *Material and Methods:* Medline, Scopus, and the Cochrane library were searched for relevant studies up to December 2020. The primary outcomes were pain intensity improvement and the incidence of RC and LHBT graft retears. Secondary outcomes were functional scores and acromiohumeral distance (AHD) improvements. *Results:* Nine studies described surgical techniques of SCR using the LHBT, and four clinical studies reported the outcomes of the technique. The mean pain intensity improved from 4.9 ± 2.3 to 1.6 ± 1.5 in terms of the visual analog scale, exceeding the minimum clinically important difference for adequate pain relief. Significant improvements were also noted in functional scores and AHD. When compared with other repair techniques for massive RC tears, i.e., the double-row repair, the transosseous-equivalent technique with absorbable patch reinforcement, and the traditional SCR with a fascia lata autograft, there were no significant differences in pain and function improvements. *Conclusion:* SCR using the LHBT is a useful treatment option for massive RC tears; it is equally effective with the traditional SCR and other established techniques. It presents numerous advantages being a safe, easy, time-saving, and cost-effective method. The only precondition for the technique is the presence of an intact LHBT. Additional clinical trials are necessary to determine which treatment is superior for treating massive RC tears, as well as to evaluate the long-term results of the technique.

## 1. Introduction

Massive rotator cuff (RC) tears remain a challenge for the shoulder surgeon due to muscle fatty infiltration and tendon retraction [1]. When treated conservatively, persistent pain and RC arthropathy may develop. On the contrary, arthroscopic repair under tension yields disappointing results with a rate of structural failures of up to 94% [2,3]. Several surgical techniques have been proposed depending on the patients’ age and the occurrence of accompanying arthritis [1]. Reverse shoulder arthroplasty is successful in older patients, especially when arthritis is present, while in younger patients, joint-preserving procedures from debridement and partial repair to patch augmentation or tendon transfers are preferred [4].

Superior capsular reconstruction (SCR) was introduced by Mihata et al. in 2007 as a possible treatment for irreparable RC tears [5]. The concept of the technique consists of the implantation of a thick fascia lata autograft to the superior glenoid rim medially and to the greater tuberosity laterally to reconstruct the superior capsular defect present in massive RC tears and restore the superior stabilizing forces [4]. This static graft acts as a restraint against the superior migration of the humeral head, thus preventing the progression of RC tear arthropathy and yielding promising functional outcomes [1]. Several authors proposed the use of acellular dermal allograft to avoid the possible autograft donor site morbidity of the original technique, although biological healing between bone and dermal allografts is not yet fully investigated [6].

An alternative to the aforementioned graft types is the long head of the biceps tendon (LHBT), which seems to overcome problems such as donor site morbidity, viability of the grafts, and additional cost of the allografts [7]. Τhe LHBT has been utilized as an augmentation tendon patch for the surgical treatment of irreparable massive RC tears [8,9]; in the case of SCR, the supraglenoid LHBT insertion is left intact, and the LHBT is fixed to the greater tuberosity with anchors. The purpose of the current study was to systematically review the literature and present the surgical techniques and clinical studies utilizing the LHBT for SCR.

## 2. Material and Methods

This systematic review was performed in accordance with the recommendations from PRISMA (Preferred Reporting Items for Systematic Reviews and Meta-Analyses) [10].

### 2.1. Literature Search

Two independent reviewers thoroughly searched Medline, Scopus, and the Cochrane Library for studies up to December 2020. The search strategy used was “((superior capsul* reconstruction) OR (superior labr* reconstruction) OR (superior capsul* repair)) AND biceps”, adjusted to each database (the asterisk symbol broadens the search by including all the words that start with the same letters). The reference lists of relevant studies were also manually searched.

### 2.2. Eligibility Criteria

Studies utilizing the LHBT for SCR and describing the surgical technique and/or clinical outcomes were included. To elaborate, only techniques in which the supraglenoid insertion of the LHBT was left intact and the LHBT was fixed to the greater tuberosity with anchors were included; techniques which included tenotomy of the LHBT at the supraglenoid insertion and augmentation of the LHBT in the RC repair were excluded, as they did not follow the concept of SCR. Animal studies, cadaveric studies, letters to the editor, and review articles were excluded.

### 2.3. Study Selection and Data Collection

The selection of the studies was performed by two independent authors after studying the abstracts and then the full-texts of the relevant records. Data were extracted by the same authors separately. Any disagreements were discussed and resolved by an additional referee. The characteristics of the studies (type, year of publication, country), as well as the study population characteristics, and the outcomes of interest were collected and interpreted.

### 2.4. Quality Assessment

For clinical studies, two reviewers critically appraised the studies for potential sources of bias. The Coleman Methodology Score was used for comparative studies (0 to 100; 100 indicating the highest quality) [11], and the checklist developed by Moga et al. was used for case series studies (0 to 18; 0–6 low quality, 7–12 moderate quality, 13–18 high quality) [12]. For studies describing surgical techniques, the adequacy of the description of the procedure details, any adjuvant procedures, and the postoperative rehabilitation was evaluated.

### 2.5. Outcome Measures

The primary outcome measures were the improvement in visual analog scale (VAS) for pain and the incidence of RC and LHBT graft retears [13]. Secondary outcomes included improvements in functional scores and improvement in the acromiohumeral distance (AHD) as the smallest distance from the inferior surface of the acromion to the superior aspect of the humeral head, evaluated either using magnetic resonance imaging (MRI), or ultrasound examination.

### 2.6. Statistical Analysis

Continuous data were reported as mean values and standard deviations, with dichotomous data as proportions. The overall improvement in VAS for pain was calculated using weights according to the studies’ sample sizes. To interpret the VAS change scores, the minimum clinically important difference (MCID) for adequate pain control of −3 (in a VAS scale from 0 to 10) was used [14,15]. Microsoft Excel version 16 and IBM SPSS (Statistical Package for Social Sciences) software version 24 were used for data analysis.

## 3. Results

### 3.1. Identification of Studies

**Figure 1:**
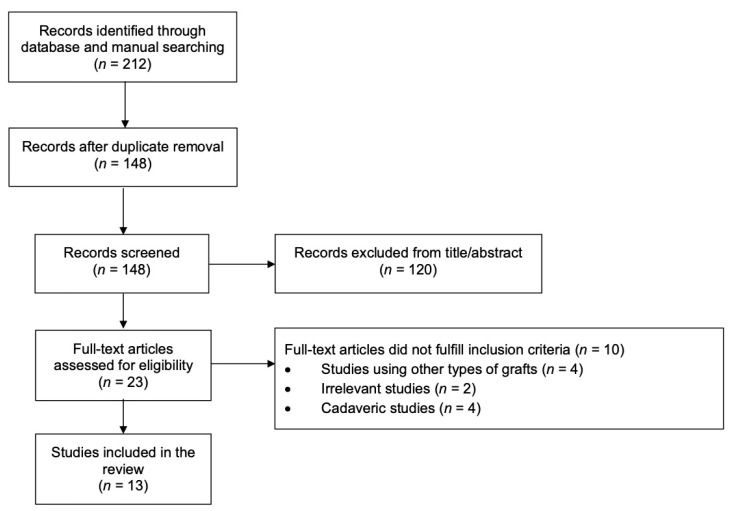
Flow diagram for study selection process.

The database search identified 212 potentially relevant records for inclusion. After the removal of 64 duplicates and 120 irrelevant records from title and abstract screening, 23 full-text articles were assessed for eligibility. Ten studies did not fulfill the inclusion criteria, leaving 13 studies for inclusion in the review (Figure 1). Nine studies described relevant surgical techniques without clinical outcomes [7,16,17,18,19,20,21,22,23], while the remaining four were clinical studies [2,24,25,26].

### 3.2. Surgical Techniques Studies’ Characteristics

Nine studies described SCR using the LHBT without clinical outcomes and the dates of publication ranged between 2017 and 2020 [7,16,17,18,19,20,21,22,23]. Four studies were conducted in Asia [16,19,21,22], three in Europe [17,20,23], one in North America [18], and one included authors both from Asia and Europe [7]. The indications of the procedure were irreparable [7,16,17,18,19,22,23] or repairable [20,21] RC tears, and most authors reported fatty infiltration up to Goutallier/Fuchs grade 4 [7,23,25,26]. Several authors reported an intact or repairable subscapularis tendon as an indication [7,17,21,23]. Contraindications were osteoarthritis of the glenohumeral joint, shoulder stiffness, deltoid muscle atrophy or axillary nerve injury, and lesions of the LHBT, i.e., severe tendinitis, partial tear >20% of its substance, and superior labrum anterior to posterior (SLAP) lesion III-IV [7,16,17,18,19,20,21,22,23].

**Figure 2:**
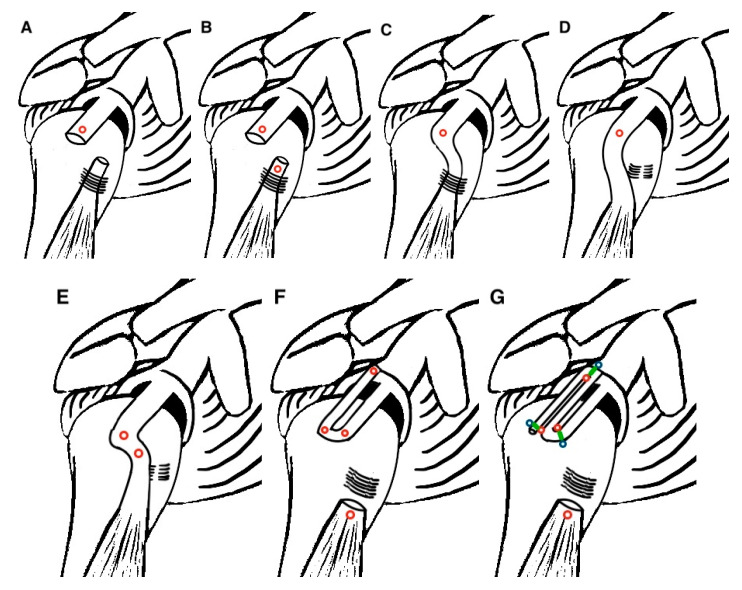
Illustration of the various LHBT (long head of the biceps tendon) management options. Red circles represent suture anchors, blue circles knotless anchors, and green lines suture tapes. (**A**) Fixation to the greater tuberosity and simple tenotomy; (**B**) Fixation to the greater tuberosity and tenodesis; (**C**) Fixation to the greater tuberosity, LHBT left intact into the bicipital groove; (**D**) Fixation to the greater tuberosity, LHBT left intact and rerouted in new groove; (**E**) Fixation to the greater tuberosity, LHBT left intact and rerouted, additional fixation of the LHBT to the anterior footprint; (**F**) double-bundle LHBT autograft and tenodesis; (**G**) triple-bundle LHBT autograft and tenodesis.

In all described techniques, the supraglenoid insertion of the LHBT was left intact and the LHBT was fixed to the RC footprint using anchors either in 30° [16,17,19,20,21] or 40° of shoulder abduction [7]. The LHBT distal to the fixation point was managed in four ways; either simple tenotomy [7,20], tenotomy with optional tenodesis in active patients [21], tenotomy with standard tenodesis [17,19], or LHBT left intact [16,18,22,23]. In the studies which reported that the LHBT was left intact, two authors resected the transverse humeral ligament, resulting in a rerouted LHBT [16,18], while the remaining two authors described either the release of the upper part of the ligament [23], or no release at all [22], keeping the LHBT in the bicipital groove. Fandridis et al. and Kim D. et al. described modifications using longer autografts of the LHBT, harvested through a separate anterior incision at the level of the inferior border of the pectoralis major, aiming for superior capsule reconstruction with double- or even triple-bundle LHBT autograft [17,19]. The various options for the management of the LHBT are illustrated in Figure 2.

A repairable tear of the RC was repaired using single- or double-row techniques including the LHBT in the repair [20,21]. For irreparable RC tears, partial repair of the remnants was performed with anchors and augmentation of the LHBT with side-to-side sutures [7,16,17,18,19,22,23]. The subscapularis was repaired separately if possible [7,17,20,23]. The key surgical steps of each technique article are presented in Table 1.

Postoperatively, most authors implemented a massive RC tear repair protocol with an abduction brace for 4–8 weeks [7,16,17,19,20,21,22], with active hand, wrist, and elbow exercises allowed from the first postoperative day. Passive and active-assisted exercises of the shoulder were initiated after the removal of the brace, and progressive active rehabilitation exercises were initiated after 8–12 weeks [16,17,18,19,20,21,22]. Strengthening exercises and overhead sports participation were allowed after 16–24 weeks [7,17,19,20,21,22].

### 3.3. Clinical Studies’ Characteristics

Two retrospective non-randomized comparative studies [2,26] and two prospective case series studies [24,25] reported clinical results of SCR using the LHBT and were published between 2018 and 2020 [2,24,25,26]. Two studies were conducted in Asia [2,24] and two in Europe [25,26]. The studies analyzed 178 participants, of which 108 underwent SCR using the LHBT. The clinical studies’ characteristics are presented in Table 2.

Barth et al. utilized SCR with the LHBT tenotomized and the stump fixed to the greater tuberosity with an anchor in 40° of shoulder abduction (Figure 2A) [2]. They compared the technique with two well-established repair techniques for massive RC tears; double-row repair, and the transosseous-equivalent technique with absorbable patch reinforcement. Chillemi et al. utilized the same technique of SCR with the LHBT in a series of patients, using two knotless anchors or two transosseous tunnels for fixation on the greater tuberosity [24]. Kim J. et al. in another series of patients performed SCR with the LHBT and left the LHBT intact, rerouting the tendon distal to the fixation point by transecting the transverse humeral ligament, creating a new tendon groove on the humeral head using a burr, and fixing the LHBT with one additional anchor to the anterior footprint in 30° of shoulder abduction (Figure 2E) [25]. Kocaoglu et al. on the other hand left the LHBT intact in the bicipital groove (Figure 2C) and proposed the use of an extra anchor to the supraglenoid insertion of the LHBT to reinforce it [26]. In their study, they compared the SCR with the LHBT with the traditional SCR with a fascia lata autograft. They fixed the LHBT to the greater tuberosity with an anchor in 40° of shoulder abduction. All studies followed the key concepts of the massive RC tear repair protocol mentioned earlier. The critical steps of the procedures followed in the four clinical articles are presented in Table 3.

### 3.4. Quality Assessment

All surgical techniques studies adequately described the main procedure details, any adjuvant procedures, as well as the postoperative rehabilitation protocol. Regarding the two comparative studies (level of evidence III), the study published by Barth et al. scored 75% and the study by Kocaoglu et al. 74% using the Coleman Methodology Score [2,26]. The remaining two case series studies (level of evidence IV) were deemed to be of high quality according to the checklist developed by Moga et al. [24,25].

### 3.5. Clinical Outcomes

The mean VAS for pain improved from 4.9 ± 2.3 to 1.6 ± 1.5 after the SCR with the LHBT, exceeding the MCID for adequate pain control. It should be noted that the VAS improvement in the study by Kim et al. did not exceed the MCID, even though the difference was statistically significant [25]. Two studies evaluated the retear rate using MRI [25,26] and one using ultrasound examination [2]. The overall retear rate was 22.2% for the RC and 20.2% for the LHBT graft. Of note, Barth et al. reported worse outcomes in the two cases of retears of the RC, while Kim J. et al. reported no significant differences in the final clinical outcomes or range of motion between retear and non-retear cases [2,25]. All functional scores and the AHD improved significantly between initial preoperative assessment and final follow-up. The improvements in clinical scores and AHD are presented in detail in Table 4.

When compared with other repair techniques for massive RC tears, i.e., the double-row repair and the transosseous-equivalent technique with absorbable patch reinforcement, all functional outcomes, range of motion, and pain VAS had similar improvements, except for the strength of the operated shoulder, where the difference was significantly better in the group undergoing SCR with the LHBT (*p* = 0.006) [2]. Moreover, the survival analysis with retear as the endpoint revealed that the median time to rotator cuff retear was longer than the other techniques [2].

Similarly, when compared with the traditional SCR with a fascia lata autograft, there was no significant difference in VAS, range of motion, and functional scores improvement, as well as in retear rates [26]. The SCR with the LHBT showed better outcomes in terms of AHD, although not statistically significant [26].

## 4. Discussion

### 4.1. Summary of Results

The present systematic review aimed to assess SCR with the use of the LHBT as an autograft. Several modifications of the surgical technique have been proposed; however, they all share the basic concept of SCR by leaving the supraglenoid insertion of the LHBT intact, and fixing the LHBT to the RC footprint using anchors. Four clinical studies assessed the clinical outcomes of the technique, reporting significant improvements in pain intensity, functional scores, and AHD between initial preoperative assessment and final follow-up [2,24,25,26]. The mean pain intensity improved from 4.9 ± 2.3 to 1.6 ± 1.5 in terms of VAS for pain, exceeding the MCID for adequate pain relief. When compared with other repair techniques for massive RC tears, i.e., the double-row repair, the transosseous-equivalent technique with absorbable patch reinforcement, and the traditional SCR with a fascia lata autograft, there were no significant differences in pain and function improvements. Notably, SCR with the LHBT resulted in increased strength of the operated shoulder compared with the double-row repair and the transosseous-equivalent technique.

### 4.2. Interpretation of the Results in the Context of the Literature

The traditional SCR matches favorably to current options for treating massive RC tears regarding clinical outcomes. SCR shows a lower retear rate compared with primary and augmented RC repairs, which reach up to a 90–100% rate [27,28]. A recent study by Neumann et al. showed equivocal short-term results with bridging RC repair using dermal matrix xenografts; however, more studies are warranted for the use of patch augmentation of massive RC tears [29]. Comparisons of SCR with latissimus dorsi tendon transfer showed larger improvements in functional scores and range of motion in patients undergoing SCR [4]. Pogorzelski et al. compared SCR with latissimus dorsi tendon transfer, and only SCR patients had statistically significant improvements in postoperative functional scores (*p* = 0.002 versus *p* = 0.161), and the mean change in abduction and flexion were −7.3° and 0.6°, respectively, in the tendon transfer group, compared to 56.0° and 21.7°, respectively, in the SCR group [30]. Finally, reverse shoulder arthroplasty achieves similar functional outcomes, but a higher complication rate than SCR (39% versus 7%, respectively) [4].

SCR with the use of the LHBT autograft yielded similar results with the traditional SCR in the direct comparison performed by Kocaoglu et al., including the retear rates (3/14 for the LHBT SCR versus 2/12 for SCR with a fascia lata) [26]. SCR graft retear rates have been reported in the literature and they range from 3.4–55% for human dermal allograft and 4.5–29% for fascia lata autografts, revealing less satisfactory results in the long-term [6,31]. Mihata et al. reported that the success of the SCR depends mainly on graft healing [31]. The intact LHBT attachment in the supraglenoid tubercle may contain sufficient vascularity and improve the graft healing rates, as well as the proprioception of the joint [19]. The LHBT incorporation in the partial RC repair also adds supplemental tissue rich in live tenocytes and fibroblasts.

Other advantages of the technique over the traditional SCR are the lower cost, the eliminated donor-site morbidity and incisions compared to other autografts, the reduced inflammatory reactions compared to allografts, and the potential decrease of infection due to the shorter operative time [7,16,20]. Moreover, it is technically easier and reproducible than SCR with a fascia lata autograft or dermal allograft, which requires a long learning curve.

Mihata et al. recommended using autografts 8 mm thick and with 6.1 cm mediolateral and 3 cm anteroposterior dimensions [5,32], while the dermal allograft used is 3.5 mm thick and the dimensions measure 4 × 7 cm, which can be adapted according to the lesion’s size and the glenohumeral anatomy [7]. The mean LHBT diameter is 6.6 mm, which is close to the proposed graft thickness and is deemed mechanically sufficient, given that the strength of the LHBT measures 32.5 ± 5.3 MPa, whereas the value for the supraspinatus tendon is only 16.5 ± 7.1 MPa [33]. The LHBT is not as wide as the fascia lata or the dermal graft and has been criticized as not being suitable for SCR because the smaller surface coverage of the humeral head may lead to its superior escape. However, recent biomechanical studies have shown that SCR with the LHBT, especially when combined with side-to-side anterior and posterior marginal repair of the RC with the LHBT, re-centers the humeral head on the glenoid, prevents the superior humeral migration, and is equivalent to a fascia lata graft [34,35,36,37].

A potential disadvantage of the technique is that the potentially inflamed LHBT has been considered a source of pain, and its use in the SCR could theoretically cause tension on the proximal insertion and continual postoperative pain [24]. However, the results of the clinical studies showed no difference in terms of postoperative pain among the compared techniques, either when the LHBT was tenotomized or rerouted, suggesting that it can be safely used as an autograft [2,24]. Another disadvantage of the technique is that the LHBT cannot be used in the presence of severe tendinitis, partial tear, SLAP lesion >II, and in the case of the extremely rare anatomic variations or absence of the tendon, and an alternative graft for SCR should be considered [38].

### 4.3. LHBT Management

Regarding the management of the LHBT distal to the fixation point on the greater tuberosity, three options have emerged in the described techniques. Advocators of the tenotomy without tenodesis argue that it is a cost-effective and simple pain-relieving method, while those of the tenodesis that simple tenotomy may result in weakness on elbow flexion and supination, cramps, and popeye-sign deformity [39]. However, the literature so far reported no difference in pain relief and functional outcome between the two techniques [39,40,41,42]. In the third option, the LHBT is left intact and is supposed to create a tenodesis effect and a downward force to the humeral head, increasing the AHD and preventing the progression to cuff tear arthropathy [18,25,26]. In certain studies, the intact LHBT was rerouted in a new groove laterally [16,18,25]. It is currently unknown whether transposing the tendon out from its groove and securing it in a nonanatomic location leads to negative effects [43]. However, there may be long-term advantages related to the downward force vector applied to the superior humeral head. 

Regarding the supraglenoid insertion of the LHBT, which was preserved by all authors, Kocaoglu et al. proposed additional fixation with an anchor [26]. They utilized the same technique as in SLAP repair and they argued that the glenoid insertion of the LHBT is not sufficiently tight because of degeneration and aging. They also proposed that the fixation of the LHBT to the greater tuberosity should be made in 40° of shoulder abduction.

### 4.4. Implications for Future Research

Additional comparative trials are necessary to determine which treatment is superior for treating massive RC tears, as well as which modification of the technique may provide improved outcomes. Moreover, high-quality research is warranted to evaluate the long-term results of the SCR with the LHBT, including the revision rates and the progression to cuff tear arthropathy. So far, the SCR with the LHBT autograft seems noninferior to the other established techniques.

### 4.5. Limitations

The present systematic review is limited by the low number of clinical studies and the low level of evidence of the included studies. This reflects the fact that the SCR with the LHBT autograft is a recently described technique. As a result, it was not feasible to conduct a meta-analysis between the techniques. Also, two studies were retrospective, which may create recall or selection bias. Consequently, future high-quality research is necessary.

## 5. Conclusions

SCR using the LHBT is a useful treatment option for massive RC tears, with comparable clinical outcomes and improvement in AHD compared with the traditional SCR and other established techniques. It presents numerous advantages to the traditional SCR, making it a safe, easy, time-saving, and cost-effective method. The only precondition for the technique is the presence of an intact LHBT. Additional clinical trials are necessary to determine which treatment is superior for treating massive RC tears, as well as to evaluate the long-term results of the technique.

## Figures and Tables

**Table 1:** Critical surgical steps in surgical techniques studies.

Study (Year)	Anesthesia	Position	Surgical Steps					
			Subacromial Decompression	Subscapularis Repair	LHBT Graft Harvesting Through Separate Incision	LHBT Fixation	Management of LHBT Distal to Fixation	RC Tear
Adrian (2020) [18]	N/R	Beach chair or lateral decubitus	Bursectomy, optional acromioplasty	N/R	No	1 Anchor to GT	Intact, rerouted	Partial repair
Boutsiadis (2017) [7]	General and interscalene block	Beach chair	Bursectomy, acromioplasty	Yes	No	1 Anchor to GT	Tenotomy	Partial repair, side-to-side to LHBT
Chiang (2019) [21]	General	Beach chair	Bursectomy, optional acromioplasty	N/R	No	1 Anchor to GT	Tenotomy or tenodesis	Repair including LHBT
Fandridis (2020) [17]	N/R	Lateral decubitus	Bursectomy, optional acromioplasty	Yes	8–12 cm sufficient for double-bundle	2 anchors to superior glenoid, 2 anchors to GT	Tenodesis	Partial repair, side-to-side to LHBT
Hermanowicz (2018) [23]	General	Beach chair	Bursectomy, optional acromioplasty	Yes	No	1 Anchor to GT	Intact	Partial repair, side-to-side to LHBT
Kim Y. (2018) [16]	General	Lateral decubitus	Bursectomy, optional acromioplasty	N/R	No	1 Anchor to GT, 1 anchor for medial LHBT to anterior footprint	Intact, rerouted	Partial repair
Kim D. (2019) [19]	General and suprascapular block	Beach chair	Bursectomy, acromioplasty	N/R	14 cm sufficient for double- or triple-bundle	2 anchors to superior glenoid, 4 anchors to GT	Tenodesis	Partial repair, side-to-side to LHBT
Kim D. (2020) [22]	General	Beach chair	Bursectomy, acromioplasty	N/R	No	1 Anchor to GT	Intact	Partial repair including LHBT
Milano (2020) [20]	General and/or interscalene block	Beach chair or lateral decubitus	Bursectomy	Yes	No	1 Anchor to GT	Tenotomy	Repair including LHBT

N/R: Not reported; LHBT: Long head of biceps tendon; GT: Greater tuberosity; RC: Rotator cuff.

**Table 2:** Summary of clinical studies’ characteristics.

Study (Year)	Intervention	Number of Patients	Males/Females	Mean Age, Years (SD)	Mean Follow-up, Months (SD)
Barth (2020) [2]	Double-row repair Transosseous-equivalent repair with absorbable patch SCR with LHBT	A. 28 B. 30 C. 24	A. 15/13 B. 19/11 C. 16/8	A. 63 (9) B. 59 (7.6) C. 60 (7)	A. 15 (2) B. 27 (5) C. 25 (2)
Chillemi (2018) [24]	SCR with LHBT	9	4/5	66.4 (3)	6
Kim J. (2020) [25]	SCR with LHBT	61	25/36	64.5 (8.2)	21.2 (range 18–27)
Kocaoglu (2020) [26]	SCR with LHBT SCR with fascia lata autograft	A. 14 B. 12	N/R	A. 64.6 (8.4) B. 62.5 (6.5)	A. 28 B. 32

N/R: Not reported; SCR: Superior capsular reconstruction; LHBT: Long head of biceps tendon.

**Table 3:** Critical surgical steps in clinical studies.

Study (Year)	Anesthesia	Position	Surgical Steps				
			Subacromial Decompression	Subscapularis Repair	LHBT Fixation	Management of LHBT Distal to Fixation	RC Tear
Barth (2020) [2]	General and interscalene block	Beach chair	Bursectomy, acromioplasty	Yes	1 Anchor to GT	Tenotomy	Partial repair, side-to-side to LHBT
Chillemi (2018) [24]	General and/or interscalene block	Beach chair or lateral decubitus	Bursectomy	Yes	2 Anchors or 2 transosseous tunnels to GT	Tenotomy or tenodesis	Side-to-side convergence to LHBT
Kim J. (2020) [25]	General	Lateral decubitus	Bursectomy, acromioplasty	Yes	1 Anchor to GT, 1 anchor for medial LHBT to anterior footprint	Intact, rerouted	Partial repair including LHBT
Kocaoglu (2020) [26]	General	Beach chair	Bursectomy, optional acromioplasty	Yes	2 anchors to superior glenoid, 2 anchors to GT	Intact	Partial repair, side-to-side to LHBT

LHBT: Long head of biceps tendon; GT: Greater tuberosity; RC: Rotator cuff.

**Table 4:** Improvement in clinical scores and acromiohumeral interval after SCR with LHBT.

	Mean Preoperative (SD)	Mean Postoperative (SD)	*p*
Barth (2020) [2]			
Pain VAS	5.2 (2)	1.4 (1.4)	<0.001
Constant Score	50 (13)	77 (10)	<0.001
ASES	45 (19)	80 (15)	<0.001
SST	4 (3)	8 (3)	<0.001
SSV	41 (22)	75 (18)	<0.001
Strength, kg	2.3 (1)	6.4 (1.6)	
Chillemi (2018) [24]			
Pain VAS	7.2	2.3	<0.01
Kim J. (2020) [25]			
Pain VAS	3.7 (2)	1.6 (1.7)	0.019
ASES	60 (19.7)	85. 2 (11.4)	<0.001
KSS	64.3 (15.5)	85.3 (11.4)	0.001
AHD, mm	7.1 (2.1)	9 (2.9)	<0.001
Kocaoglu (2020) [26]			
Pain VAS	8.5 (3.5)	1.4 (0.8)	0.001
ASES	46.2 (16.2)	85.2 (12.4)	0.005
Quick DASH	52.5 (12.8)	12.6 (18)	0.012
AHD, mm	7 (1.5)	10.2 (2.5)	0.04

SCR: Superior capsular reconstruction; LHBT: Long head of biceps tendon; VAS: Visual Analog Scale; ASES: American Shoulder and Elbow Surgeons scale; SST: Simple Shoulder Test; SSV: subjective shoulder value; KSS: Korean Shoulder Scale; Quick DASH: Quick Disabilities of the Arm, Shoulder and Hand score; AHD: Acromiohumeral distance.
